# A real world example of coverage with evidence development in Australia - ipilimumab for the treatment of metastatic melanoma

**DOI:** 10.1186/s40545-018-0131-4

**Published:** 2018-02-13

**Authors:** Hansoo Kim, Samantha Comey, Karl Hausler, Greg Cook

**Affiliations:** Bristol-Myers Squib, Level 2/4 Nexus Court, Mulgrave, VIC 3170 Australia

## Abstract

**Background:**

Australian Government subsidisation of ipilimumab for the treatment of patients with metastatic melanoma was conditional on the sponsor entering a ‘managed entry scheme’ to assess the 2-year overall survival rate in metastatic melanoma patients who received ipilimumab in the first year of Pharmaceutical Benefits Scheme listing.

**Methods:**

All unresectable stage IIIc / IV metastatic melanoma patients treated with at least one dose of ipilimumab therapy in Australia from the PBS listing date to a time point 12 months later (i.e. from 1-Aug-2013 to 31-Jul-2014) were invited to participate. Overall survival at 2 years post treatment initiation was measured, with Cox regression analysis used to examine the relationship between survival and patient baseline characteristics.

**Results:**

The evaluable population (910 patients) was on average 63.3 years old, male (70.1%) and treated in a public hospital (64.4%) in an urban area (76.5%). The majority of patients were treatment naïve (63.3%), did not have brain metastases (71.1%), and were classified as ECOG performance status 0 or 1 (90.4%). The 2 year overall survival rate was conservatively calculated to be at least 23.9% and potentially as high as 34.2%. A significant difference in overall survival at 2 years was demonstrated across the categories of ECOG performance status (*p* < 0.0001), M-status (*p* = 0.0005) and treatment status (*p* = 0.0114). No statistical difference in survival rate was observed when examining brain metastases vs no brain metastases (*p* = 0.2622), treatment at private vs public hospitals (*p* = 0.7601) nor treatment in the urban vs rural setting (*p* = 0.5048).

**Conclusions:**

The 2 year overall survival rate for all patients receiving PBS subsidised ipilimumab in Australia from the first year Pharmaceutical Benefits Scheme cohort is estimated to be between 23.9% and 34.2%, which is higher than the 23.5% observed in the key ipilimumab registrational trial. Results and learnings from the ipilimumab ‘managed entry scheme’ illustrate that early access with the promise of future evidence to confirm a medicine’s cost-effectiveness can work, but needs to be carefully considered, constructed and managed.

## Background

Significant developments in technology and evidentiary profiling, together with increased patient and clinician advocacy for early access to innovative medicines that promise major therapeutic gain, has led to varying degrees of registration and reimbursement system evolution across the globe.

From a regulatory perspective, agencies around the world are responding to evolving clinical trial design and clinical trial data demonstrating significant advantages over existing options for patients with serious or life-threatening diseases. The US Food and Drug Administration (FDA) now has four potential pathways for rapid evaluation of new medicines - namely the Priority Review pathway, the Breakthrough Therapy pathway, an Accelerated Approval pathway and a Fast Track Designation pathway [1]. Similarly the European Medicines Agency (EMA) allows for Accelerated Assessment and/or Conditional Marketing Authorisation for innovative medicines that target a disease for which no treatment is available or that provide patients with a major therapeutic advantage over existing therapies [2].

In Australia, reform to the regulatory process to allow earlier registration in some circumstances is currently being implemented. A review of Medicines and Medical Devices Regulation (MMDR review) was announced by the Australian Government in October 2014 and undertaken by an Expert Panel through 2015 [3]. The MMDR review noted the increasing globalisation of the pharmaceutical and medical devices industries and the rapid pace of innovation, and accordingly made a number of recommendations as to how to position the Australian Therapeutics Goods Administration (TGA) to respond to these trends in the future. One such recommendation related to potential expedited pathways for the registration of new medicines in certain circumstances (Priority Review and Provisional Approval pathways). While it has been mooted that these expedited pathways could allow medicines to reach Australian patients up to 2 years earlier than under the current framework, this would only be possible if the Australian reimbursement system aligned with the TGA on how to deal with the likely increase in clinical (& economic) uncertainty attached to the Provisional Approval pathway. To not do so risks the gap between TGA registration and Pharmaceutical Benefits Scheme (PBS) reimbursement lengthening.

The PBS has been a pivotal component of Australia’s healthcare system since 1948, providing affordable access to necessary and lifesaving medicines for all Australians. While the PBS has attracted both controversy and scrutiny, it has served the Australian community well, often cited globally as the gold standard with respect to provision of universal access to medicines [4, 5]. System evolution in response to medical and economic challenges has been the key to the success and longevity of the PBS over the last seven decades. While a relatively recent process change to allow for regulatory and reimbursement review to be conducted in parallel has reduced the gap between TGA registration and PBS listing [6], a PBS system capable of dealing with greater clinical and/ or economic uncertainty will be required in order to align with the greater demand for early access to medicines and the evolving clinical trial model and regulatory systems.

Coverage with evidence development (CED) is one such policy option that may need to be adopted more consistently and aligned to the TGA Provisional Approval pathway, in order to truly facilitate earlier PBS access to vital medicines in Australia.

Trueman et al. [7] define CED as “restricted coverage for a new technology in parallel with targeted research where the stated goal of the research or data collection is to provide definitive evidence for the clinical or cost-effective impact of the new technology”. While CED has existed as a concept for over two decades, it must be noted that uptake and success of previous CED programs is varied. Differences across disease areas, medicines and clinical / economic uncertainties has seen CED study design, outcomes, and the application of conditions remain neither straightforward nor standardised [8, 9].

While the first publically acknowledged example of CED in Australia was specific to bosentan for the treatment of pulmonary arterial hypertension in 2004 [10] a formal mechanism for PBS reimbursement with the promise of future data was not introduced until January 2011. Initially termed Managed Entry Scheme (MES) and now referred to as Managed Access Program (MAP) [11, 12]. Only a small number of medicines have been identified as potential candidates [Table 1] – and none to date have publically reported on the benefits, risks and learnings of such arrangements.

**Table 1:** Medicines identified as potential MES candidates since introduction of formal MES policy in January-2011 [35]

Medicine^a^	MES
Ipilimumab for metastatic melanoma (2012)	• Pay for performance with rebates payable should 2 year overall survival rates in real world clinical practice in Australia not align with clinical trial data
Ivacaftor for cystic fibrosis (2014)	• Pay for performance with rebates applicable for patients subsequently assessed as non-responders
Eculizumab for atypical haemolytic uraemic syndrome (2014)	• Pay for performance with rebates applicable for patients who do not achieve an agreed clinical outcome over an agreed time periods
Trametinib for metastatic melanoma (2014)	• Pay for performance with rebates applicable should trametinib fail to deliver claimed benefits
Crizotinib for non-small cell lung cancer (2014)	• Pay for performance with rebates applicable should crizotinib fail to deliver claimed benefits
Pembrolizumab for metastatic melanoma (2015)	• PBS list with provision for future clinical trial evidence to support a potential price increase
Nivolumab for non-small cell lung cancer (2016)	• PBS list with provision of future evidence to confirm effectiveness of nivolumab in NSCLC patients ≥75 years of age

^a^Medicines considered by the PBAC between January 2011 and November 2016

Ipilimumab is one medicine that listed on the PBS via a MES arrangement. This paper focuses on providing details and experiences from the ipilimumab MES in an effort to add to the current debate as to how best to provide Australian patients earlier access to innovative medicines in areas of high clinical need.

Ipilimumab, a monoclonal antibody that works to activate the immune system by targeting CTLA-4 [13], was the first immunotherapy listed on the PBS for the treatment of metastatic melanoma [14, 15]. The PBS listing for ipilimumab was conditional on the sponsor providing future overall survival (OS) evidence to support/ confirm the proposed cost-effectiveness of this medicine in the treatment of Australian patients with metastatic melanoma.

The geographical location of Australia coupled with a large Caucasian population means that the incidence of melanoma in Australia is among the highest in the world. It is estimated that 13,941 new cases of melanoma skin cancer will be diagnosed in Australia in 2017, translating to an age–standardised incidence rate of 50 cases per 100,000 persons [16] – almost quadruple that observed in Europe (13.2 per 100,000) [17].

Until recently, survival for patients with metastatic melanoma in Australia was very poor as available funded treatments (radiation, surgery and chemotherapy using dacarbazine) were largely ineffective [18]. However the potential for improved survival for Australian patients increased recently with the inclusion of innovative immunotherapies on the Australian Government’s subsidised reimbursement list – the PBS. Ipilimumab was the first immunotherapy available via the PBS, listing on August 1, 2013, with pembrolizumab and nivolumab (highly specific programmed death-1 [PD-1] immune checkpoint inhibitors), following in 2015 [14, 15, 19, 20].

Historically, medicines used for the treatment of metastatic melanoma were largely ineffective. Korn et al. [21] reported a historical 2-year overall survival of 8.1% based on a pooled meta-analyses of 42 clinical trials in metastatic melanoma. The ipilimumab clinical trial (NCT00094653) used for initial regulatory approval [22] in Australia demonstrated an overall survival (OS) rate of 23.5% at 2 years (ITT analysis) [13]. As ipilimumab is a novel medicine compared to traditional chemotherapies there was some uncertainty expressed by Australian reimbursement authorities (the Pharmaceutical Benefits Advisory Committee, PBAC) as to how trial results would translate into real world clinical practice. The PBAC were accepting of ipilimumab’s manageable and controllable safety profile and also considered that the key clinical trial delivered evidence of a plateau effect with ipilimumab treatment [14]. However, while the PBAC acknowledged that there was some evidence that the effect was durable, they also noted that the magnitude of the benefit remained a source of uncertainty with modelled survival curves at the end of the time horizon driven by extremely small patient numbers [14]. The PBAC, although concerned about the cost-effectiveness of ipilimumab if the claimed survival gain was not observed in practice, recommended the listing of ipilimumab for metastatic melanoma, subject to a risk-sharing arrangement to confirm the predicted cost-effectiveness of ipilimumab. Specifically, the PBAC requested the implementation of a mechanism to verify the anticipated OS benefits of ipilimumab in real world clinical practice in Australia [14]. Post the PBAC recommendation the sponsor and representatives from the PBAC and Department of Health met and discussed the framework of the ipilimumab MES (MAP).

It was agreed that in order to address the concerns of the PBAC, OS at 2-years was to be assessed in the ‘real-world’ setting for all patients initiated on ipilimumab during the first full year of PBS listing. The results would then be compared to the 2 year OS data from the key ipilimumab clinical trial (NCT00094653), with the sponsor to rebate the cost of difference in performance between observed versus predicted OS benefits of ipilimumab should observed OS be less than that seen in the key clinical trial. Safety parameters were not included as this was not identified as a major source of uncertainty for the PBAC.

## Methods

All unresectable stage IIIc/ IV metastatic melanoma patients treated with at least one dose of ipilimumab therapy in Australia from the PBS listing date to a time point 12 months later (i.e. from 1-Aug-2013 to 31-Jul-2014) were invited to participate.

All medical oncologists initiating patients on ipilimumab during the 1-year enrolment period were asked to register through a website and complete online training prior to prescribing ipilimumab. Upon receipt of the PBS listing date, all identified treating oncologists received a letter notifying them of the listing date and of the requirement to collect the ipilimumab survival rate at 2 years post treatment initiation. The letter also contained instructions on how to prescribe ipilimumab and how to register patients on the website. Requirements of the program were also disseminated through professional channels, face-to-face interactions, and through medical information.

Registration of patients on the website required patient consent in order to perform the 2 year follow up. Personal details such as name, date of birth and address were not captured and instead each patient was given a unique reference identification number. Ordering of ipilimumab by the pharmacist triggered dispatch of the medicine and receipt by the pharmacist no later than the following working day. The date of first dispatch was used as proxy for the first infusion date.

Participating sites and treating oncologists were prompted via email to review the patient’s medical record in order to provide the survival status of their patients at 2 years post first infusion date. A second e-mail was automatically sent if the physician had not entered the data within 2 weeks of the 2 year anniversary. Follow up phone calls or visits were performed if responses were not received within 2 weeks of the second e-mail.

Outcome (alive, dead or lost to follow-up) was recorded along with date of outcome. Patients with unknown status were recorded as unconfirmed outcomes status. As the sponsor was required to provide a report to the PBAC no later than 60 business days after the end of Year 3 (21-October- 2016), a cut-off date of 23-Sep-2016 was set for the data gathering, allowing for 4 weeks of follow-up and resolution of queries.

In line with the agreed MES (MAP) requirements, a 2 year OS estimate was calculated, including number of patients alive, dead or lost to follow up at the two year time point. Descriptive statistics (e.g. age, weight, gender) and an analysis of the representativeness of the patients (proportion providing informed consent, rural vs urban, public vs private institutions) was also conducted. It is important to note that no data was collected on dosing, treatments received concomitantly or post ipilimumab, nor occurrence of adverse events.

The data was initially reported using summary statistics for each demographic variable: age, body weight, gender, hospital type (private/public) and community setting (rural/urban). Age and body weight was split up into three categories in order to examine the survival and mortality rates. Standard Kaplan-Meier survival analysis [23] was also performed, as was Cox regression modelling [23] in order to examine the relationship between mortality and baseline characteristics: age, bodyweight, Eastern Cooperative Oncology Group (ECOG) performance status [24], metastatic (M) stage [25], whether patients had prior brain metastases, whether patients had been treated with systemic therapy prior to receiving ipilimumab, whether patients were treated in a private hospital vs public hospital and whether patients were treated in a rural or urban setting. Data analyses were performed using SAS 9.2 on a Windows 7 platform.

## Results

**Fig. 1:**
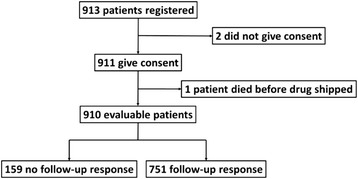
Patient disposition

From 1-Aug-2013 to 31-Jul-2014, a total of 913 Australian metastatic melanoma patients were registered on the website. Of these, two patients declined to participate and one patient passed away before the medicine was dispatched, leaving a total of 910 evaluable patients. The outcome status for 159 patients was not obtained at the time of data base lock, leaving 751 patients with evaluable follow-up response (refer to Fig. 1 for patient disposition).

Review of the baseline characteristics of the evaluable population (Table 2) revealed that two-thirds of patients were aged over 60 with the average age being 63.3. The cohort population was predominantly male (70.1%) with the mean body weight recorded as 80.9 kg. The vast majority of patients had an ECOG performance status of 0 or 1 (i.e. 90.4% were categorised as active or restricted) with the remaining 9.6% categorised as ECOG performance status 2 or above (i.e. self-care capable, limited self-care capable or disabled). With regards to M stage disease classification, 74.6% of the evaluable population were deemed to be M1c, 17.2% were classified as M1b, with the remaining 8.2% assessed as M1a. A total of 28.9% of patients had experienced previous brain metastases, with 63.3% of the evaluable cohort recorded as treatment naïve in the metastatic melanoma setting. A majority of patients lived in an urban setting (76.5%) and received treatment via the Australian public hospital system (64.4%). Retrieval of outcome data was comparable across all baseline disease characteristics and demographic variables, ranging from 77.4% to 100%.

**Table 2:** Baseline characteristics and demographics

	Summary statistic	Retrieval of 2 Year OS data
Age (years)		
Mean (sd)	63.3 (13.0)	
< 40 years	53 (5.8%)	41 (77.4%)
40–60 years	256 (28.1%)	211 (82.4%)
> 60 years	601 (66.0%)	499 (83.0%)
Body weight (kg)		
Mean (sd)	80.9 (17.8)	
< 70 kg	246 (27.0%)	199 (80.9%)
70–90 kg	412 (45.3%)	349 (84.7%)
> 90 kg	252 (27.7%)	203 (80.6%)
Gender		
Female	271 (29.9%)	221 (81.6%)
Male	635 (70.1%)	528 (83.3%)
ECOG performance status		
Active	351 (38.7%)	293 (83.5%)
Restricted	468 (51.7%)	65 (90.3%)
Self-care capable	73 (8.1%)	379 (81.0%)
Limited self-care capable	13 (1.4%)	11 (84.6%)
Disabled	1 (0.1%)	1 (100%)
M Status		
M1a (distant skin, subcutaneous or nodal metastases)	74 (8.2%)	63 (85.2%)
M1b (lung metastases)	156 (17.2%)	124 (79.5%)
M1c (All other visceral metastases or any distant metastases with elevated serum LDH)	676 (74.6%)	562 (83.3%)
Previous brain metastases		
No	644 (71.1%)	517 (80.3%)
Yes	262 (28.9%)	232 (88.9%)
Treatment naïve		
No	333 (36.7%)	278 (83.7%)
Yes	573 (63.3%)	471 (82.2%)
Hospital		
Private	301 (35.6%)	242 (80.1%)
Public	544 (64.4%)	456 (84.0%)
Community		
Rural	199 (23.5%)	169 (84.9%)
Urban	647 (76.5%)	529 (81.9%)

**Fig. 2:**
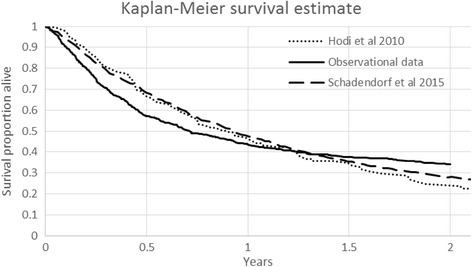
Kaplan-Meier survival estimate*

At the 2 year anniversary post treatment initiation, the survival rate in patients who provided consent was 23.9% (218/911). Among evaluable patients, 24.0% (214/910) were confirmed alive at or beyond the 2 year anniversary, 52.3% (476/910) were confirmed deceased prior to the 2 year anniversary and 6.3% (57/910) were confirmed lost to follow-up by the treating physician. There was no response from the treating physician regarding the status of the remaining 17.5% (159/910) patients. Depending upon how patients without 2 year OS data were incorporated into the evaluable patients’ calculations, the real-world 2 year OS rate could potentially be as high as 29.0% (218/751, removing patients for which there was no response), with the Kaplan-Meier analysis (taking censoring into account) estimating the OS to be 34.2% at 2 years (Fig. 2).

* The Kaplan-Meier curves for overall survival from the pivotal clinical trial reported by Hodi et al. [13] and Schadendorf et al. [26] for ipilimumab are also displayed in Fig. 2 as reference curves.

**Fig. 3:**
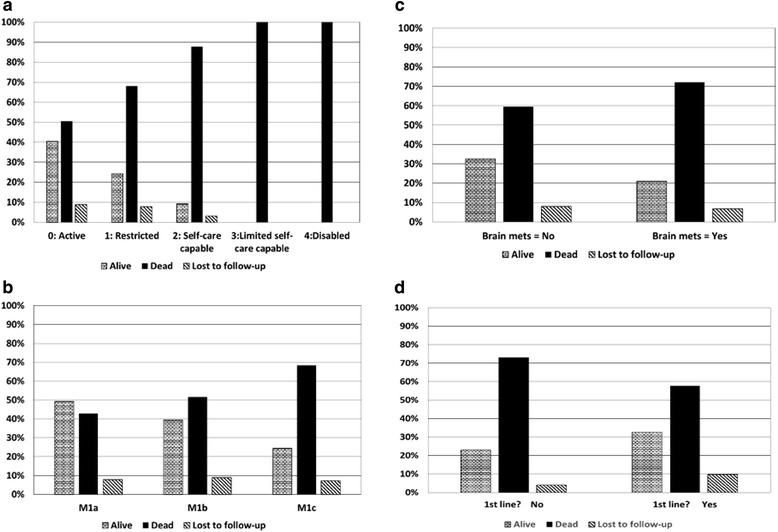
Survival by demographic variables. **a** Survival by ECOG performance status, **b** Survival by metastatic status, **c** Survival by brain metastases and **d** Survival by treatment naïve/experienced

Age, weight and gender were not shown to be predictors of survival (Table 3). The results do however suggest a potential relationship between ECOG performance status and survival. Forty-one percent of patients classified as ECOG 0 were alive 2 years post ipilimumab treatment initiation. This figure decreased to 24% for patients classified as ECOG 1, 9% for ECOG 2 patients and 0% for ECOG 3–4 patients (Fig. 3a). Statistical analysis demonstrated that there is a significant difference in OS across ECOG performance status (*p* < 0.0001; Table 3).

**Table 3:** Cox regression analysis

Parameter tested	*p*-value Hazard ratio [95% CI] (if relevant)
Age	*p* = 0.1644
Body weight	*p* = 0.4476
Gender	*p* = 0.1750
Female vs Male	1.163 [0.94; 1.45]
ECOG performance status	*p* < 0.0001
1: Restricted vs 0: Active	1.76 [1.42; 2.18]
2: Self-care capable vs 0: Active	3.00 [2.13; 4.22]
3: Limited self-care capable vs 0: Active	6.91 [3.64; 13.11]
4: Disabled vs 0: Active	629.65 [56.44; 7024.33]
M Status	*p* = 0.0005
M1a vs M1c	0.54 [0.43; 0.81]
M1b vs M1c	0.66 [0.36; 0.82]
Previous brain metastases	*p* = 0.2622
No vs Yes	0.88 [0.71; 1.10]
Treatment naive	*p* = 0.0114
No vs Yes	1.29 [1.06; 1.57]
Hospital	*p* = 0.7601
Private vs Public	0.965 [0.77; 1.21]
Community	*p* = 0.5048
Urban vs Rural	1.07 [0.88; 1.31]

Likewise, when examining results according to M-staging of the disease, a significant drop in survival was seen with increasing M-stage. A two-year OS rate of 49.2% was recorded for patients with M-stage = M1a. This decreased to 39.5% for patients with M-stage = M1b disease and decreased again to 24.4% for patients with M-stage = M1c (Fig. 3b). Statistical analysis revealed that there is a significant difference between in OS across M-stage status (*p* = 0.0005; Table 3).

Patients with prior brain metastases were demonstrated to have a higher mortality rate when compared to those without brain metastases (72.0% vs 59.5%; Fig. 3c), although this difference did not reach statistical significance (*p* = 0.2622; Table 3).

The survival rate for patients who had been previously treated with systemic therapy in the metastatic setting was lower (23.0%) when compared to patients who were treatment naïve (32.5%; Fig. 3d), with this difference reaching statistical significance (*p* = 0.0114; Table 3).

No statistical difference in OS was observed for patients treated in a public vs a private hospital, nor for patients treated in a rural vs urban setting (public vs private hospital *p* = 0.7601, urban vs rural setting *p* = 0.5048; Table 3).

## Discussion

Increasing patient and clinician demand for early access to medicines that promise significant clinical advances, together with an evolving clinical trial model, has led to the establishment of fast-track regulatory pathways for breakthrough therapies in areas of high clinical need.^1^

In order for changes to the Australian regulatory system to truly translate to earlier access to innovative medicines for Australian patients, a reimbursement pathway capable of dealing with increased levels of uncertainty is also required. While acknowledging that this may not be possible in every instance, one potential method of dealing with uncertainty is to provide initial coverage with the promise of future clinical trial or real-world data [27, 28]. While the Australian reimbursement system allows for this through a PBAC MES mechanism (now also referred to as MAP) [29], uptake has been limited (Table 1) and public details virtually non-existent. This lack of transparency has been cited as a major drawback because it precludes public understanding of the ways in which decisions about initial and continued funding are made [12]. A greater understanding of the pros and cons of earlier funding coverage under the current system would assist with any evolution required to improve the MES (MAP) system and/or better align with the proposed MMDR provisional TGA registration process. It is important to note that the aim of this paper was to present results for the ipilimumab case and for a more fulsome discussion and debate in this important area of policy evolution, the perspectives of clinicians, Government, industry groups and patients are also needed.

Results from a phase III randomised controlled trial (NCT00094653) demonstrated significant OS benefits associated with ipilimumab treatment that are maintained beyond 2 years in greater than 23% of patients [13]. This data led to ipilimumab being approved for registration in Australia on July 5, 2011, for treatment of patients with unresectable or metastatic melanoma. This data was also reviewed for Government reimbursement by the PBAC on three separate occasions in July 2011 [30], March 2012 [31] and November 2012 [14], before listing on the PBS August 1, 2013.

In their review of each of the applications for reimbursement, the PBAC expressed concern about the cost-effectiveness of ipilimumab should the claimed survival gain not be observed in clinical practice. The November 2012 meeting saw the PBAC recommend PBS listing for ipilimumab on the proviso that a MES risk-sharing arrangement be established with the sponsor to develop a mechanism by which to verify the OS benefits of ipilimumab in real-world clinical practice in Australia [14]. The sponsor developed a protocol that would determine the 2 year OS rate specific to all Australian metastatic melanoma patients initiated on ipilimumab in the first full year of PBS listing.

Results from all Australian patients initiated on ipilimumab in the first year of PBS listing demonstrate a 2 year OS rate of at least 23.9% and potentially as high as 34.2%. This real-world OS rate compares favourably with the key ipilimumab clinical trial data (NCT00094653) demonstrating an OS rate of 23.5% at 2 years [13], and supports the original claim by the sponsor that ipilimumab is a cost-effective option in the Australian setting [32].

The results from the experiences of this Australian MES provide important insights on a number of levels. Firstly, the real world evidence support the OS results derived from the key ipilimumab registrational study (NCT00094653); secondly, the data results confirm that disease state is a predictor of survival rate; and thirdly, the proactive collaboration between the sponsor and Australian payer on this project demonstrates that pragmatic solutions can be found for any remaining clinical and/or economic uncertainty, thereby benefitting patients through earlier access to subsidised medicines.

While results for this project support the use of provisional funding to allow earlier access to innovative medicines in areas of high clinical need, it does not necessarily translate that this is the solution every time. Indeed, as cited by Garrison et al., “*It is critical for policy makers to recognise the benefits, limitations and methodological challenges in using RW data, and the need to consider carefully the costs and benefits of different forms of data collection in different situations*” [27].

With specific regards to the ipilimumab PBS MES (MAP), there were a number of significant learnings that could be utilised in the consideration and construction of future provisional reimbursement arrangements. To fully gauge the success or otherwise of this MES (MAP) and potential for improvements in the future, perspectives of the Australian payer, clinicians, patients and industry would also be of benefit.

The inherent inability of real world data to directly mirror the strong internal validity of a clinical trial is a significant risk to being able to answer the research question. In the case of the ipilimumab PBS MES, there was likely to be an initial cohort of patients that were extremely unwell due to the lack of an effective PBS listed therapy prior to ipilimumab PBS listing. In general the disease level of patients who received ipilimumab in the real world life cohort could be considered worse, with 9.4% having an ECOG status of 2 (Self-care capable) or 3 (Limited self-care capable) at baseline compared to 1.2% observed in the key ipilimumab clinical trial data (NCT00094653). In addition more patients in the real world cohort had previous brain metastases compared to the trial population (28.9% vs 11.4%). This potential negative impact on the OS numbers may have been countered by the availability of medicines not listed on the PBS and used post ipilimumab (e.g. dabrafenib, trametinib, pembrolizumab & nivolumab via compassionate access programs). The potential to tease out the impact of these factors on the 2 year survival rate is not possible within the context of this report. With hindsight the authors would have allowed for the capture of additional details with regards to the patient’s treatment history prior to and/or post ipilimumab therapy. A recommendation would be that any future design of MESs (MAPs) need to explicitly define the research question and factor in potential unintended consequences associated with treating patients in the real world setting and changing practices in the disease area of interest.

Setting up of the MES (MAP) was both resource intensive and costly, and to do so on a regular basis and across multiple jurisdictions is likely not seen by sponsor companies as sustainable. While the conditions of the program in relation to obtaining 2 year survival data were clearly stated at the time of clinician/patient enrolment, unconfirmed outcomes status was approximately 40% at the 2 year anniversary of the program (end of July 2016). Significant effort and resources were required to gather the full set of data presented in this manuscript. Despite this, 17.5% of patients remained classified as having an “unconfirmed outcome”. The general feedback from physicians was that it was difficult to locate patients’ medical records to determine survival status without exact personal information such as date of birth. The authors strongly advise that any future MESs (MAPs) establish robust and comprehensive reporting systems as a key component of the undertaking. Not only will it save significant time at the back end of the project, but will allow for greater confidence in the results.

While the ipilimumab MES (MAP) was established as a pragmatic solution to delivering access to Australian patients in the face of data uncertainty, it was raised and implemented as a last resort option. The PBAC had rejected the sponsor’s reimbursement submission 3 times over 2 years in the lead-up to the MES (MAP). Earlier discussions as to the potential value and viability of a MES (MAP) for ipilimumab could have led to an earlier PBS listing. With the likely imminent introduction of provisional registration in Australia via the MMDR review, discussions specific to provisional reimbursement/ MAPs ideally need to occur prior to PBAC submissions and /or after a first-time PBAC rejection.

## Conclusion

In conclusion, the advocacy, clinical trial and regulatory environment has moved significantly in recent years towards the concept of early access for breakthrough medicines in areas of high clinical need. While the Australian reimbursement system has investigated MES (MAP) as a way to provide Australian patients early access to innovative medicines in the face of clinical and/or economic uncertainty, experiences are not extensive and publically available learnings are limited. The ipilimumab MES (MAP) described in this paper is the first such example to be published and not only illustrates that such arrangements can work successfully, but also provides valuable learnings. Clearly more work in this space is required, especially with the recent advent of a provisional TGA registration pathway. Additional transparency from other Australian MESs (MAPs), together with learnings from the patient, clinician, industry and payer’s perspective are also needed to ensure the environmental push for earlier access to breakthrough medicines can be fully explored.

## Abbreviations

CED: Coverage with Evidence Development
ECOG: Eastern Cooperative Oncology Group
EMA: European Medicines Agency
FDA: Food & Drug Administration
MAP: Managed Access Program
MES: Managed Entry Scheme
MMDR: Medicines and Medical Devices Regulation
OS: Overall Survival
PBAC: Pharmaceutical Benefits Advisory Committee
PBS: Pharmaceutical Benefits Schedule
RW: Real World
TGA: Therapeutic Goods Administration

## Footnotes

1 It is relevant to note that recent reviews of the US [33] and Canadian [34] fast track regulatory programs revealed that not all medicines delivered significant advances.
